# The diagnosis of leptospirosis complicated by pulmonary tuberculosis complemented by metagenomic next-generation sequencing: A case report

**DOI:** 10.3389/fcimb.2022.922996

**Published:** 2022-10-04

**Authors:** Jichan Shi, Wenjie Wu, Kang Wu, Chaorong Ni, Guiqing He, Shilin Zheng, Fang Cheng, Yaxing Yi, Ruotong Ren, Xiangao Jiang

**Affiliations:** ^1^ Department of Infectious Disease, Wenzhou Central Hospital, The Dingli Clinical College of Wenzhou Medical University, Wenzhou, China; ^2^ Institute of Innovative Applications, MatriDx Biotechnology Co., Ltd, Hangzhou, China; ^3^ Institute of Infectious Diseases, Center for Disease Control and Prevention, Wenzhou, China; ^4^ Foshan Branch, Institute of Biophysics, Chinese Academy of Sciences, Beijing, China

**Keywords:** leptospirosis, mNGs, early diagnosis, pulmonary tuberculosis (PTB), co-infection

## Abstract

Leptospirosis is a zoonotic infection caused by the pathogenic *Leptospira*. Leptospirosis is transmitted mainly through contact with contaminated rivers, lakes, or animals carrying *Leptospira*. Human leptospirosis has a wide range of non-specific clinical manifestations ranging from fever, hypotension, and myalgia to multi-organ dysfunction, which severely hampers the timely clinical diagnosis and treatment of leptospirosis. Therefore, there is an urgent clinical need for an efficient strategy/method that can be used for the accurate diagnosis of leptospirosis, especially in critically ill patients. Here, we report a case of a 75-year-old male patient with clinical presentation of fever, cough, and diarrhea. Initial laboratory tests and a computed tomography (CT) scan of the chest suggested only tuberculosis. The patient was finally diagnosed with pulmonary tuberculosis (PTB) combined with leptospirosis by sputum Xpert MTB RIF, epidemiological investigations, and delayed serological testing. Furthermore, through metagenomic next-generation sequencing (mNGS) of clinical samples of cerebrospinal fluid (CSF), urine, plasma and sputum, the causative pathogens were identified as *Mycobacterium tuberculosis* complex and *Leptospira* spp. With specific treatment for both leptospirosis and tuberculosis, and associated supportive care (e.g., hemodialysis), the patient showed a good prognosis. This case report suggests that mNGS can generate a useful complement to conventional pathogenic diagnostic methods through more detailed etiological screening (i.e., at the level of species or species complex).

## Introduction

Leptospirosis is an acute bacterial septic zoonosis caused by *Leptospira* infection (El-Tras et al., 2018). Leptospirosis usually occurs in tropical and subtropical regions, and has been reported sporadically in some epidemic areas of China, mainly in the warm and rainy provinces located in southern and central regions (Zhang et al., 2020). Human infection with pathogenic *Leptospires* presents with asymptomatic, or widespread non-specific symptoms, i.e., from the first stage of sepsis (manifested by fever, chills, vomiting, diarrhea, etc.) to the second stage of severe multisystem damage (e.g., renal and liver failures) (Haake and Levett, 2015; De Brito et al., 2018) with potential life-threatening symptoms in severe cases. Due to the wide range of non-specific presentations described above, leptospirosis is often misdiagnosed, especially when comorbidities with overlapping presentations are encountered (Budihal, 2014; Nhan et al., 2016; Suppiah et al., 2017; Lau et al., 2018). Metagenomic next-generation sequencing (mNGS) can theoretically sequence/detect any nucleic acids from biological samples, including pathogenic microorganisms and is therefore considered to be a very important complementary diagnostic method for pathogenic microorganisms (Gu et al., 2019). Here, through conventional pathogenic testing methods and mNGS co-diagnosis, we report a case of leptospirosis presenting as acute renal failure with tuberculosis. To our knowledge, this would be the first clinical case report of leptospirosis complicated by tuberculosis detected by mNGS.

## Case presentation

### Clinical presentations

A 75-year-old male patient from Yongjia county (Zhejiang province, China) visited our hospital [i.e., Wenzhou Central Hospital (WCH)/The Second Affiliated Hospital of Shanghai University] on 16 November 2021 (defined as day 0) with the manifestation of fever accompanied by expectoration for 6 days and diarrhea for 5 days.

The patient had visited a local clinic and received oral medication (the detail was unknown) 6 days ago due to fever and paroxysmal cough with yellow sticky phlegm. The patient had a maximum body temperature of 39.0°C and felt no chills then (i.e., 6 days ago). One day later, the patient started to have an episode of watery diarrhea with a stool frequency of four to five times per day, which is accompanied by pains in bilateral thighs and shanks. He then visited the Second Affiliated Hospital of Wenzhou Medical University on the same day (i.e., 5 days ago), where he was administered with piperacillin sodium and tazobactam sodium [4.5 g, intravenous (I.V.) infusion every 8 h (*quaque octa hora*, q8h)], levofloxacin [0.5 g, I.V. infusion one a day (*quaque die*, qd)], and fluid infusion.

**Figure 1:**
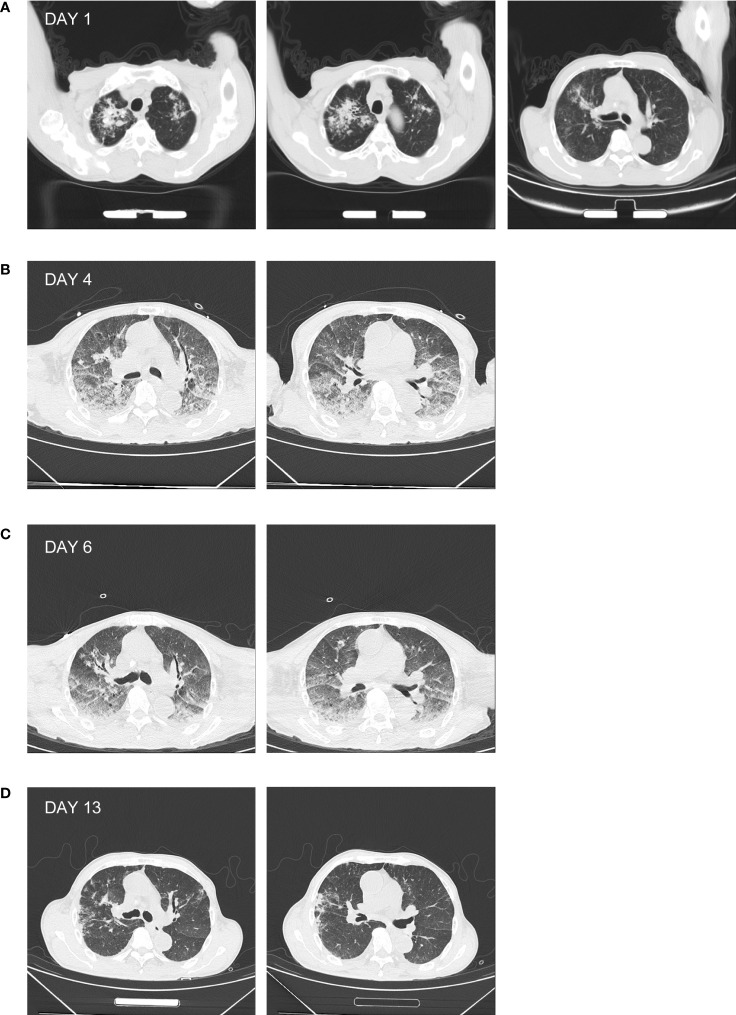
Chest CT of the patient. **(A)** The chest CT prior to admission showing high-density patchy and streak-like shadows in the lung, with unclear margin. **(B)** The chest CT of day 4 showing enhanced patchy and nodular high-density shadows in the lung, associated with diffused ground-glass opacities. **(C)** The chest CT of day 6 showing the relief of the patchy high-density shadow. **(D)** The chest CT of day 13 showing the absorption of the patchy high-density shadow.

A chest CT on day 0 showed binary pulmonary infection, which was in suspicion of pulmonary tuberculosis (PTB) (**Figure 1A**). Given the pulmonary infection and acute renal failure, the patient was admitted to our hospital the next day and subsequently transferred to the infectious intensive care unit (ICU) of our hospital for further diagnosis and treatment.

### Physical examination

On admission, the patient had an ear temperature of 37.2°C, a pulse rate of 83 beats per minute, a blood pressure of 100/54 mmHg, and a respiratory rate of 18 breaths per minute. At that time, he was conscious and mentally normal. Physical examination showed that both of his pupils were round and equal in size (3 mm in diameter). There was no jaundice, scleral icterus, spider angioma, or palmer erythema observed. The patient’s neck was supple, trachea was at the midline of the neck, and the patient had no jugular vein engorgement. Abnormal breathing sounds was auscultated in bilateral lungs of the patient, and occasional moist rale in the right lower lobe. In addition, the patient had normal heart rhythm and no pathological murmurs of valves on auscultation. His belly was soft, and there was no (rebound) tenderness as revealed by palpation. The liver and spleen were not palpable. There was no leg edema. The bilateral pathological reflexes were negative.

### Laboratory examination

The result of initial laboratory examination (day 1) was as follows: white blood cell count (WBC), 11.43 × 10^9^ cells/L; neutrophils % (neut. %), 0.924%; hemoglobin (HB), 88 g/L; platelets (PLT), 86 × 10^9^ cells/L; C-reactive protein (CRP), 165.53 mg/L; total protein, 52.8 g/L; albumin, 26.6 g/L; bilirubin, 22.1 μmol/L; and creatinine, 464 μmol/L (**Table 1**).

**Table 1:** Laboratory results.

Hospitalized date	WBC^a^(×10^9^/L)	HB^a^(g/L)	ALB^a^(g/L)	PLT^a^(×10^9^/L)	CRP^a^ (mg/L)	PCT^a^(μg/L)	PT^a^(s)	TBiL^a^(μmol/L)	Cr^a^(μmol/L)	LDH^a^(U/L)	CK^a^(U/L)
Day 1 (17 November 2021)^b^	11.43	88	26.6	86	165.5	89.87	14.8	22.1	464	352	464
Day 2	8.0	77	20.7	71	154.1	85.64	14.1	9.2	574	356	424
Day 3^c^	9.9	99	26	96	161.1	29.06	14.9	10.6	342	557	212
Day 4	14.7	99	27.7	134	130.9	13.6	13.6	18.2	176	704	245
Day 6^d^	6.9	72	21.3	154	43.2	3.81	13.4	8.0	398	612	458
Day 9	6.2	66	24.1	231	10.8	1.19	12.4	7.9	185	493	138
Day 11	6.6	65	24.8	247	6.8	0.69	12.2	7.8	151	454	77
Day 14	6.4	67	26.5	235	2.3	0.45	11.3	6.4	106	286	78
Day 21	4.1	70	28.5	119	12.7	–	–	5.3	91	–	–

^a^WBC, white blood cells; HB, hemoglobin; ALB, albumin; PLT, platelets; CRP, C-reactive protein; PCT, procalcitonin; PT, prothrombin time; TBil, total bilirubin; Cr, creatinine; LDH, lactic dehydrogenase; CK, creatine kinase. ^b^The day of admission was defined as day 1. ^c^The day performing Xpert MTB RIF (positive). ^d^The day obtaining the mNGS result of CSF, urine, and plasma (the mNGS result of sputum was obtained on day 8).

### Diagnostic procedure and treatment

Anti-infectious treatment was empirically administered with sulperazone (2.0 g, I.V. infusion, q8h) and moxifloxacin (0.4 g, I.V. infusion, qd) on day 1, combined with supportive care for relieving cough, reducing sputum, and regulating intestinal flora and fluid infusion. The patient still manifested with persistent fever, chest distress, and tachypnea. Oxygen was then supplied at a rate of 6 L/min using an oxygen mask to maintain an oxygen saturation of 95%. The patient had less urine and progressively elevated creatinine. He was then given methylprednisolone [40 mg, I.V. infusion *bis in die* (bid)] on day 2 to enhance the anti-inflammatory effect and reduce pulmonary exudation. Continuous renal replacement therapy (CRRT) was simultaneously applied. On day 3, the patient produced blood-tinged sputum. Xpert MTB RIF using sputum identified the presence of rifampicin-sensitive *Mycobacterium tuberculosis* complex (MTBC) (data not shown). In association with the chest CT result (**Figure 1A**), the diagnosis of PTB was confirmed. The therapeutic strategy was then altered to rifampicin [0.45 g, by mouth (*per os*, P.O.) every morning (*quaque mane*, qm)], isoniazid tablets (0.3 g, P.O., *qm*), ethambutol tablet [0.75 g, P.O., *ter* in week (tiw)], and pyrazinamide tablets (1.5 g, P.O., tiw). After CRRT, the level of serum creatinine decreased during day 2 and day 3. However, the patient became restless and delirious at the night of day 3. Since he was unable to communicate with, tolerate being in bed, or cooperate to the treatment, CRRT was forced to be terminated and sedation was applied.

**Figure 2:**
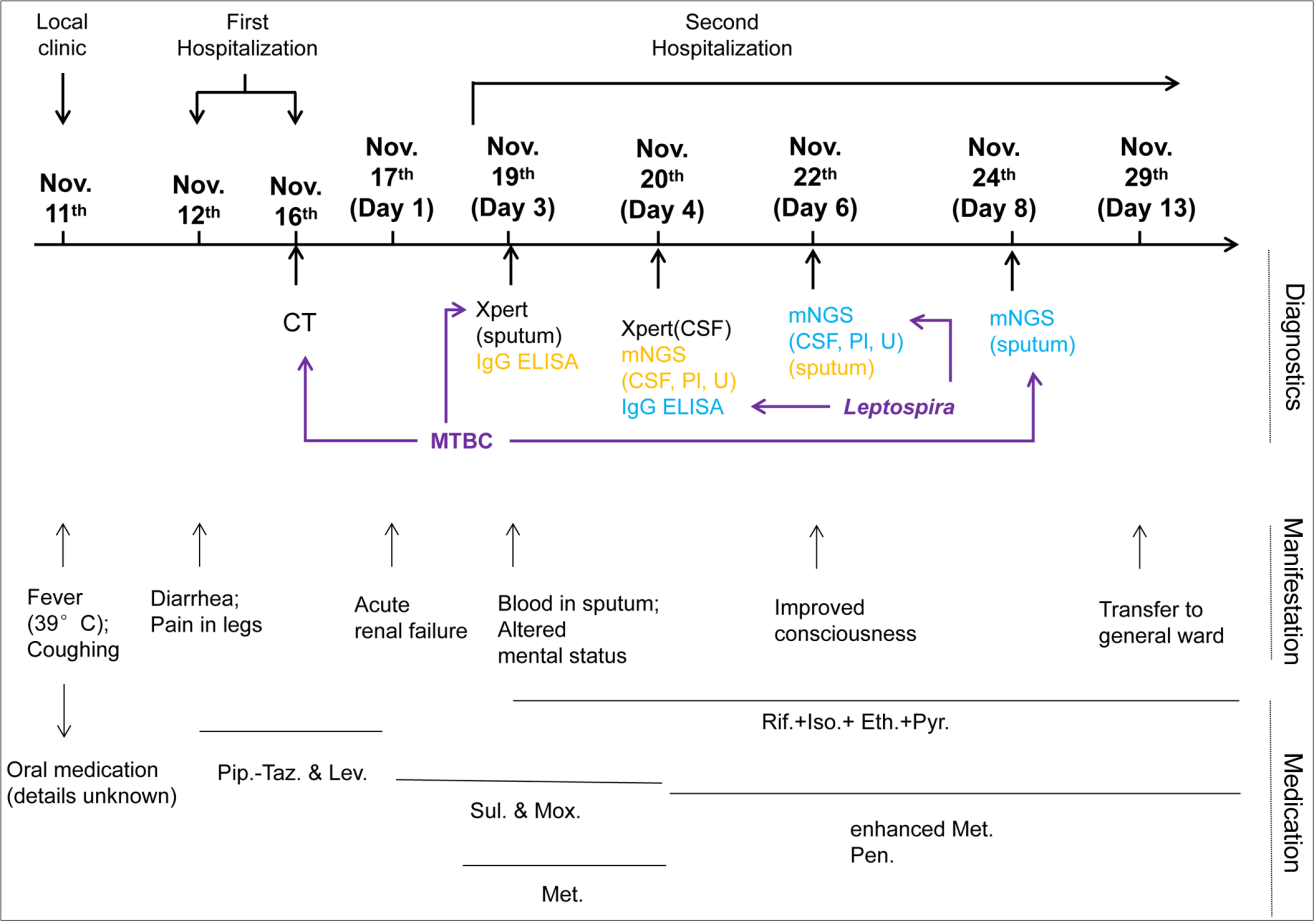
Clinical course of the patient during hospitalization. The schematic shows the timeline from the onset of symptoms (11 November 2021) to transferring to the general ward (29 November). Major events, including diagnostics, manifestations, and medication during the course, are indicated. Sample collection for diagnostics is indicated in yellow, and the dates of corresponding reports available are shown in blue. Diagnostic examinations used for pathogen identification are highlighted by purple arrows. CSF, cerebrospinal fluid; Pl, plasma; U, urine; Pip.-Taz., Piperacillin sodium and tazobactam sodium for injection; Lev., Levofloxacin hydrochloride injection; Sul., sulperazone; Mox., moxifloxacin hydrochloride and sodium chloride injection; Met., methylprednisolone; Rif., rifampicin; Iso., isoniazid; Eth., ethambutol; Pyr., pyrazinamide; Pen., penicillin.

On day 4, lumbar puncture was conducted with a CSF opening pressure of 50 mmH_2_O. CSF examination was as follows: WBC, 2 × 10^6^ cells/L; protein, 0.30 g/L; chloride, 127 mmol/L; and glucose, 5.2 mmol/L. There was neither *Cryptococcus neoformans* nor MTBC (*via* Xpert MTB RIF) detected. The samples of CSF, urine, and plasma prepared on day 4 were subjected to mNGS, by which the DNA sequences of *L. interrogans* (without MTBC) were detected with read numbers of 4, 9, and 2, respectively (reported on day 6; **Table 2**, **Supplementary Figures 1A–C**). This was in accordance with the positive screening for *Leptospira* in blood samples of the patient that were collected on day 3 and detected using the Serion ELISA classic *Leptospira* immunoglobulin G (IgG) kit by the Wenzhou Center for Disease Control and Prevention (reported on day 4 after samples being sent for mNGS). Therefore, the diagnosis of leptospirosis was verified. In combination with the heightened inflammation in bilateral lungs shown by the chest CT screen on day 4 (**Figure 1B**) as well as mental changes of the patient developed during the administration of sulperazone and moxifloxacin, the Jarisch–Herxheimer reaction (JHR) and leptospirosis-induced encephalopathy were of high suspicion. Accordingly, the dosage of methylprednisolone was increased to 80 mg from 40 mg, I.V., bid; sulperazone and moxifloxacin were replaced with penicillin (800,000 IU, I.V., q8h; the initial dose was 400,000 IU) from day 4. On day 6, the patient’s consciousness improved, so the sedation was gradually terminated (see **Table 1** for detailed laboratory tests). The chest CT of day 6 showed decreased infection foci and less amount of pleural effusion in the lung, in comparison to that of day 4 (**Figures 1B, C**). To further confirm the result of Xpert MTB RIF and CT, the sputum of the patient was also prepared for mNGS on day 6, from which the DNA sequences corresponding to MTBC (without *L. interrogans*) were detected with a read number of 4 (reported on day 8; **Table 2**, **Supplementary Figure 1D**). The mNGS result of sputum was consistent with the Xpert MTB RIF result of day 3. On day 13, patchy shadows with high density were roughly absorbed as revealed by chest CT (**Figure 1D**); thus, the patient was transferred to the general ward of the infectious disease department for subsequent treatment. The detailed clinical course of the patient during hospitalization is shown in **Figure 2**.

**Table 2:** Pathogens detected by mNGS.

Sample type	Genus	Species	Number of reads	RPM^d^	Relative abundance (%)
CSF^a^	Leptospira	*Leptospira interrogans*	4	0.3	0.07
Urine	Leptospira	*Leptospira interrogans*	9	0.74	0.06
Sputum	Mycobacterium	MTBC^b^	4	0.26	< 0.01
Plasma^c^	Leptospira	*Leptospira interrogans*	2	0.06	4.65

^a^CSF, cerebrospinal fluid. ^b^MTBC, Mycobacterium tuberculosis complex (the mNGS pipeline reports the species group (i.e., MTBC) rather than the detailed species due to their high homology). ^c^Cell-free DNA was used for metagenomic next-generation sequencing. ^d^RPM, reads per million mapped reads.

### Metagenomic NGS

After sequential operations, including the sample preparation of microbial cell wall disruption *via* bead beating (for CSF and urine, not for plasma), DNA extraction and library construction (PCR-free) using an NGS Automatic Library Preparation System (Hangzhou Matridx Biotechnology Co., Ltd., Zhejiang, China) (Luan et al., 2021), NGS on an Illumina NextSeq 550Dx (single end, 50 bp), and the data processing of removing short reads (<35 bp), low-quality and low-complexity reads, and high-quality sequencing data were generated. The generated data were then separately aligned to a human-specific database constructed from *Homo sapiens* sequences in NCBI nucleotide (nt) databases (for eliminating human sequences) using bowtie2 v2.3.5.1 (Langmead and Salzberg, 2012), and a manual-curated microbial genome database for taxonomy classification using kraken2 (confidence = 0.5) (Wood et al., 2019) and bowtie2 v2.3.5.1 (Langmead and Salzberg, 2012). The microbial genome database contained 11,704 viral genome sequences, 11,162 bacterial genome sequences, 229 parasite genomes, and 1,324 fungal genomes relevant to human infectivity. Moreover, the reads classified to *L. interrogans* or MTBC were subsequently aligned to a relevant microbial genome (i.e., *L. interrogans* or MTBC) using BLASTN v2.10.1+ (Zhang et al., 2000) for further validation (**Supplementary Figure 1D**).

## Discussion

Leptospirosis is a zoonotic disease caused by pathogenic bacteria of the genus *Leptospira*. Animals infected with *Leptospira*, such as rats, pigs, and cattle, can excrete the bacillus in their urine and contaminate water and soil over a long period of time (Schneider et al., 2013; Haake and Levett, 2015). Humans are infected with *Leptospira* primarily through direct or indirect contact with animals carrying *Leptospira* or contaminated rivers or lakes. In China, at least 80% of *Leptospira* infections occur in rice-growing areas, particularly in the Yangtze, Pearl, and Lancang River basins. Approximately 85% of leptospirosis cases occur between July and December each year, with a peak in September (Zhang et al., 2012), which also coincides with the rice sowing or harvesting period.

The patient reported in this case was from a humid and river-rich rice-growing area (part of the Yangtze River basin) in southern China (i.e., Yongjia county, Zhejiang Province, China) and had an onset and admission in November, information that is consistent with the region/seasonal pattern of leptospirosis prevalence (Zhang et al., 2012). In terms of exposure history, the patient had a history of water exposure 13 days prior to admission (3 November) in the Yongjia Mountains, which are located in the leptospirosis epidemic area. Considering the epidemiological features and the non-specific manifestations of fever, cough, diarrhea, and myalgia, the patient was clinically suspected to be infected with *Leptospira*. Subsequently, the results of anti-*Leptospira* IgG antibody testing in the patient’s blood samples and mNGS testing of the patient’s CSF, urine, and plasma verified the clinical suspicion of leptospirosis infection as described above. Leptospirosis affects the lungs mainly in the lower lung region (Tsang and File, 2008; Viswanathan and Iqbal, 2015), and focal shadows associated with infection were also present in the upper lobe of the patient’s lung, which, in combination with the mNGS results, confirmed that in addition to leptospirosis, the patient also had PTB, and the patient’s acute respiratory distress symptoms were also partially caused by PTB. Subsequently, the antibiotic treatment strategy was changed to address both leptospirosis and PTB, and the patient’s clinical symptoms were promptly relieved.

Cases of leptospirosis with other infectious comorbidities, such as dengue (Wijesinghe et al., 2015; Paul, 2022), malaria (Wilairatana et al., 2021), chikungunya (Nhan et al., 2016; Cardona-Ospina et al., 2018), and scrub typhus (Watt et al., 2003; Borkakoty et al., 2016; Vikram et al., 2020), have been increasingly described in tropical and subtropical regions, especially in the overlapping endemic regions. Co-occurrences of leptospirosis and tuberculosis in humans, however, are less frequently reported (Viswanathan and Iqbal, 2015). Our case was diagnosed with leptospirosis *via* genus-specific IgG-ELISA and PTB *via* Xpert MTB RIF, and further complemented with mNGS, which detected the species-level etiology of leptospirosis (i.e., *L. interrogans*), besides detecting MTBC and excluding other potential etiologies. The species-level clarification of leptospirosis enables the retrospective epidemic investigation of the genus *Leptospira*, which contains multiple pathogenic species (Lane and Dore, 2016). To our knowledge, under the circumstance of mNGS, this is the first reported case of leptospirosis complicated with tuberculosis.

During empirical chemotherapy against infection, JHR was induced in our case. JHR commonly occurred in patients with spirochetal infections when they accept treatments with antibiotics (Tsuha et al., 2016; Butler, 2017; Guerrier et al., 2017). The cause of JHR might be related with abundant toxins suddenly released from spirochetes that are being inactivated by antibiotics, especially potent anti-spirochetal antibiotics. JHR is basically characterized by fever and exacerbation of skin rashes; in rare cases, acute respiratory distress, alterations in consciousness, and severe renal failure may occur (Butler, 2017). In a retrospective study carried out in China, 16 out of 1,125 patients suffering from syphilis developed JHR after being treated with penicillin (Li et al., 2012). Another study of 262 patients from New Caledonia and Futuna with confirmed leptospirosis indicated that an average of 21% of patients receiving amoxicillin treatment developed JHR (Guerrier et al., 2017). Variations in occurrences of JHR is possibly related to patients’ sensitivity towards antibiotics, toxins, and/or therapeutic criteria for different spirochetal species. The patient in our case was initially treated with sulperazone and moxifloxacin after admission, then he manifested dysphoria, delirium, and heightened inflammation in bilateral lungs but without meningeal irritation.

It is also obvious that pathogenic microorganisms involved in coinfections may induce similar clinical manifestations including fever, cough, headache, and myalgia, especially during early infection stages (Bark et al., 2011; Md-Lasim et al., 2021). In fact, the overlapping spectrum of manifestations might not only be difficult for clinicians to distinguish between leptospirosis and its comorbidities, but might also be hard to differentiate from general infectious diseases, such as influenza and pneumonia. For example, chest radiographs of leptospirosis-invaded lungs usually display consolidations, a ground glass appearance, a crazy-paving pattern, or inflammatory foci in bilateral lungs, which is also frequently observed in lungs of other respiratory tract infection patients (Marchiori et al., 2008; De Wever et al., 2011). Thus, misdiagnoses and underdiagnoses frequently occur. Independent of manifestation-based diagnoses, mNGS identifies microorganisms solely based on their sequence information, which provides it with great opportunities to unveil rare or even newly emergent pathogenic microorganisms as well as those involving coinfections or diseases with synonymous clinical symptoms, especially in comparison with conventional diagnostics (Wang et al., 2019; Chen et al., 2021; Zhao et al., 2021). For example, a novel pathogenic strain of pseudorabies virus (hSD-1/2019) was for the first time identified from the CSF samples of four acute encephalitis patients with the application of mNGS (Liu et al., 2021). In a prospective study conducted by Xing et al. (2021), mNGS had been further confirmed as a powerful tool for accurate and quick diagnosis of cerebral aspergillosis, a rare and life-threatening infection that often presents with variable and non-specific symptoms, with a specificity of more than 85%, while results from cranial magnetic resonance imaging and diagnostic markers [e.g., (1→3)-β-D-glucan and GM] either were non-specific or had low sensitivities. Moreover, apart from the leptospirosis–tuberculosis coinfection introduced in this paper, mNGS had also been reported for diagnoses of the initial occurrence and the follow-up relapse of leishmaniasis in an HIV-coinfected case, in which the patient on admission presented with nonsynonymous symptoms of fatigue and splenomegaly, and cured after a parasite-resistant therapy (Song et al., 2021).

Once penetrating/infecting host *via* mucous membranes or compromised skin, *Leptospira* could disseminate systematically through the hematogenic route (De Brito et al., 2018). Since it preferentially colonizes at the proximal tubules of hosts’ kidneys, *Leptospira* might be shed out from the urinary tract (De Brito et al., 2018). MTBC, on the other hand, predominantly infects humans *via* inhaling MTBC-containing aerosol droplets. Because of its respiratory-prone pathogenic characteristics, MTBC mainly affects lungs (i.e., PTB, roughly more than 80%), while in less frequent circumstances, it could also affects other organs (i.e., extrapulmonary tuberculosis, 15%–20%) (Baykan et al., 2022). Here, in our case, MTBC was only detected in sputum in both Xpert MTB RIF and mNGS, whereas *L. interrogans* was detected in CSF, urine, and plasma, but not in sputum (**Table 2**). The mutually exclusive detection of different pathogens from different kinds of clinical samples, as exemplified in **Table 2**, highlighted that more than one kind of samples might be essential for a comprehensive diagnosis if patients do not have good prognosis due to more than one pathogen with different pathogenic characteristics. Furthermore, the detection of *L. interrogans* in CSF, urine, and plasma (**Table 2**) suggested the systematic infection of *L. interrogans* in our patient.

The pathogenic mechanism of leptospirosis remains to be fully clarified. It is known that the toxin-mediated injury of blood capillaries and the highly activated immune responses of the host are mainly involved in the development of leptospirosis (Wagenaar et al., 2009). Lysins and lipopolysaccharide of *Leptospira* stimulate monocytes and macrophages, which then express more responsive inflammatory cytokines (e.g., TNF-α, IL-6, and IL-10) (Dorigatti et al., 2005). Leptospirosis-induced kidney damages include interstitial nephritis, necrosis of renal tubular epithelial cells, and rupture of partial tubular basement membranes (Yang, 2007; Yamaguchi et al., 2018). Therefore, in addition to the treatment against *Leptospira* and the approach to stabilize hemodynamics, the usage of glucocorticoid may also be beneficial for prognosis (Muthuppalaniappan et al., 2018). Continuous hemodialysis can be one of the essential strategies for eliminating endotoxins, inflammatory mediators, and metabolic wastes, as well as regulating internal environment, managing blood volume, and nutrition support.

In conclusion, (1) the outbreak of leptospirosis has significant regional and seasonal preferences, and clinicians should pay close attention to patients’ epidemiological history and be alert to the possibility of leptospirosis; (2) China is a high-burden country for TB; therefore, once cases that could not be explained by a single pathogen/disease are encountered, comorbidity should be taken into consideration to reduce the possibility of misdiagnosis and missed diagnosis; (3) mNGS, as a novel detection method, could assist the diagnosis of leptospirosis timely; and (4) continuous hemodialysis is beneficial to the prognosis of patients suffering from leptospirosis with renal failure complicated with tuberculosis, in addition to treatment for both leptospirosis and tuberculosis.

## Data availability statement

The datasets presented in this study can be found in NCBI with the SRA accession PRJNA827932.

## Ethics statement

The studies involving human participants were reviewed and approved by the board of Ethics at Wenzhou Central Hospital (WCH)/The Dingli Clinical College of Wenzhou Medical University. The patients/participants provided their written informed consent to participate in this study. Written informed consent was obtained from the individual(s) for the publication of any potentially identifiable images or data included in this article.

## Author contributions

JS and XJ are the primary physicians who provided diagnosis and treatment of the patients. JS, WW, KW, and RR collected and analyzed clinical and sequencing data. CN, GH, SZ, FC, and YY prepared some paragraphs of the manuscript in Chinese. JS, WW, KW, and RR wrote the manuscript. All authors have read and approved the final version of the manuscript.

## Conflict of interest

Authors WW, KW, YY, and RR are employed by MatriDx Biotechnology Co., Ltd.

The remaining authors declare that the research was conducted in the absence of any commercial or financial relationships that could be construed as a potential conflict of interest.

## Publisher’s note

All claims expressed in this article are solely those of the authors and do not necessarily represent those of their affiliated organizations, or those of the publisher, the editors and the reviewers. Any product that may be evaluated in this article, or claim that may be made by its manufacturer, is not guaranteed or endorsed by the publisher.

## Supplementary material

Supplementary Figure 1 Schematic illustration of reads mapping. **(A–C)** The mapping of *L. interrogans*-specific reads from CSF **(A)**, urine **(B)** and plasma **(C)** against a *L. interrogans* genome (strain FMAS_AW1 chromosome, complete genome, NCBI Reference Sequence: NZ_CP039283.1). **(D)** The mapping of MTBC-specifc reads from sputum against a *Mycobacterium tuberculosis* (*Mtb*) genome (strain KIT87190 chromosome, complete genome, NCBI Reference Sequence: NZ_CP007809.1). The vertical blue bar indicates the individual mapping of each specifc read. The genomic locations of two *L. interrogans*-specific reads from plasma are too close to be distinguished from the mapping plot (i.e. panel **C**).

## Abbreviations

CT, computed tomography; PTB, pulmonary tuberculosis; mNGS, metagenomic next-generation sequencing; CSF, cerebrospinal fluid; WCH, Wenzhou Central Hospital; I.V., intravenous; q8h, *quaque octa hora*; qd, *quaque die*; ICU, intensive care unit; WBC, white blood cell count; neut.%, neutrophils %; HB, hemoglobin; PLT, platelets; CRP, C-reactive protein; bid, *bis in die*; CRRT, continuous renal replacement therapy; MTBC, *Mycobacterium tuberculosis* complex; tiw, *ter* in week; JHR, Jarisch-Herxheimer reaction; nt, nucleotide; IgG, immunoglobulin G.
