# Supplementary figures and images for: The diagnosis of leptospirosis complicated by pulmonary tuberculosis complemented by metagenomic next-generation sequencing: A case report
Source: Front Cell Infect Microbiol. 2022 Oct 4;12:922996. doi: 10.3389/fcimb.2022.922996 (PMC9577070; doi:10.3389/fcimb.2022.922996)

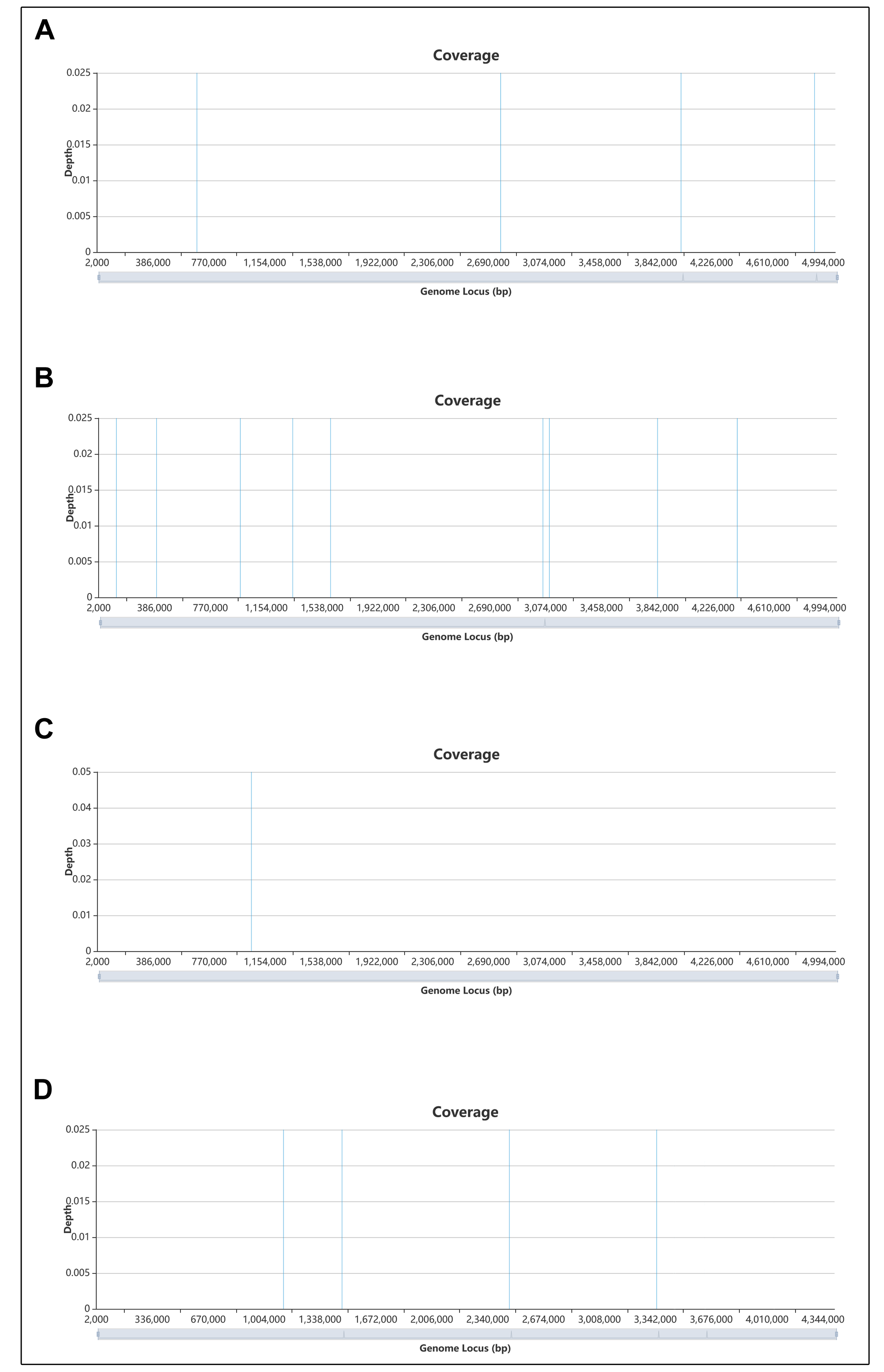

Supplement: Supplementary Figure 1 — Schematic illustration of reads mapping. (A–C) The mapping of L. interrogans-specific reads from CSF (A), urine (B) and plasma (C) against a L. interrogans genome (strain FMAS_AW1 chromosome, complete genome, NCBI Reference Sequence: NZ_CP039283.1). (D) The mapping of MTBC-specifc reads from sputum against a Mycobacterium tuberculosis (Mtb) genome (strain KIT87190 chromosome, complete genome, NCBI Reference Sequence: NZ_CP007809.1). The vertical blue bar indicates the individual mapping of each specifc read. The genomic locations of two L. interrogans-specific reads from plasma are too close to be distinguished from the mapping plot (i.e. panel C). [file Image_1.tif]
